# A prospective study for the examination of peripheral blood smear samples in pediatric population using artificial intelligence

**DOI:** 10.55730/1300-0144.5982

**Published:** 2025-03-27

**Authors:** Elif Habibe AKTEKİN, Mert Burkay ÇÖTELİ, Ayşe ERBAY, Nalan YAZICI

**Affiliations:** 1 Department of Pediatrics Division of Pediatric Hematology-Oncology, Dr. Turgut Noyan Application and Research Center, Başkent University, Adana, Turkiye; 2 Mantiscope Medical Devices Ltd, Ankara, Turkiye

**Keywords:** Pediatric hematology, prediagnostic tools, artificial intelligence, blood cell disorders, peripheral blood smear

## Abstract

**Background/aim:**

Peripheral blood smear (PBS) and bone marrow aspiration are gold standards of manual microscopy diagnostics for blood cell disorders. Nowadays, data-driven artificial intelligence (AI) techniques open new perspectives in digital hematology. This study proposes an AI learning technique for the classification of blood cells over PBS samples while increasing the sensitivity and specificity rates of the experts as a decision support system of a prediagnostic tool.

**Materials and methods:**

The methodology of this study comprises three steps for the creation of an effective learning technique for blood cell disorders. First is the digitization of PBS samples in 100x optical-digital magnification using Mantiscope which is a cloud-based slide scanner system. The second is collection of pediatric hematology experts’ annotations and the last one is data augmentation to increase the data variation and size. The data consists of 372 individuals, an approximate number of 12,000 annotated images with 500,000 blood cell objects. A subjective test is also performed to observe the interobserver variability.

**Results:**

We measured sensitivity and specificity for 28 cell types for the resulting decision support system. We obtained sensitivity 98% for myeloblast, 94% for basophil and 90% for lymphoblast, specificity 99% for basophil, eosinophil, monocyte, hypersegmented neutrophil, band neutrophil and reactive neutrophil in leukocyte subtypes. When erythrocyte measurements were evaluated, it was found that the sensitivity was 93% for normoblast, 81% for target cell and pencil cell, 80% for sickle cell, specificity was 99% for normoblast, pencil cell, echinocyte, and sickle cell.

**Conclusion:**

It is observed that sensitivity and specificity greater than 90% can be obtained for some specific cell types with this clinical study. It is seen that data augmentation increases the effectiveness of the learning method in terms of leukocytes by improving the measurement metrics. This could be a valuable technique to evaluate acute leukemias and hemolytic disorders.

## 1. Introduction

Blood cell disorders, i.e. leukemia, lymphoma, and anemia are common diseases. The diagnostic process of these diseases may contain different stages such as complete blood count with differential (CBC-D), peripheral blood smear (PBS), bone marrow aspiration (BMA), or genetic tests [1]. Several structural problems, such as high analysis costs, lack of labor resources, and occupational errors, may prevent early diagnosis. Although the popularity of PBS for hematology practice in the immunophenotyping era has diminished, it will continue to be an essential tool because it is cheap, easy to prepare, and examine. Despite CBC-D as another frequently used basic test, it cannot replace morphological findings obtained by PBS, which guides physicians [2]. Current diagnostic processes conducted worldwide require complex laboratory equipment, consumables, and trained personnel-time. Besides, these processes may differ depending on intra- and interobserver differentials [3]. It is also not practical to examine the whole slide. Given these limitations, a PBS test in which the entire sample is analyzed and more objective evaluations are made will soon provide results closer to ideal.

Artificial intelligence (AI) techniques aim to create decision support systems while helping physicians increase their sensitivity and specificity for the diagnostics. The first usage of AI in hematology starts with the examination of laboratory tests to assist physicians in education and diagnostics workflows for acute leukemia patients [4]. While these knowledge-based systems were created with the old type of machine learning (ML) techniques, recent advances in the technology offer deep learning (DL) based object detection and classification, which are dependent on data volume and consistencies. Therefore, the main motivation becomes collecting a large variety of consistent annotated data and assisting the neural network (NN) models in training.

DL-based techniques as examples for the field of hematology can be revealed to standardize the PBS and BMA evaluation tests [5]. While these studies only measure the recognition probability for the individual blood cells, overall measurements are required to define the percentages for blood cell types in PBS slides. Blood cell-specific diseases are also analyzed for prediagnostics with DL techniques, and they are proposed for analyzing samples for lymphoblastic leukemia automatically [6–8]. Segmentation of blood cells is also crucial for the detection networks to create a highly sensitive algorithm. In the means of segmentation, algorithms extract the circular region of the blood cell for object classification. A segmentation algorithm before neural networks (NN) is proposed in a study and accuracy greater than 90% is observed [9]. A standard Complete Blood Count (CBC) laboratory practice concentrates on WBC differentials. Some studies in the literature concentrate on WBC differentials with the difference in their color, nucleus, and cytoplasm when compared with the red blood cells [10]. With the known standard for the examination of PBS samples, it is also required to analyze RBC and platelets. Some DL-based techniques concentrating on sickle cells reveal an accuracy greater than 90% for detection [11]. Our main objective is to output the CBC with all cell types with PBS digital images.

A DL-based blood cell morphology analyzer is needed to create a decision support system for the identification of blood cell abnormalities. It can be used as a prediagnostic tool that would create abnormality labels associated with specific diseases. The aim of this study is to develop an image-based cell morphology analyzer that would be used by experts in initial and survey-based evaluation.

## 2. Methods

### 2.1. Data collection and annotation

**Figure 1:**
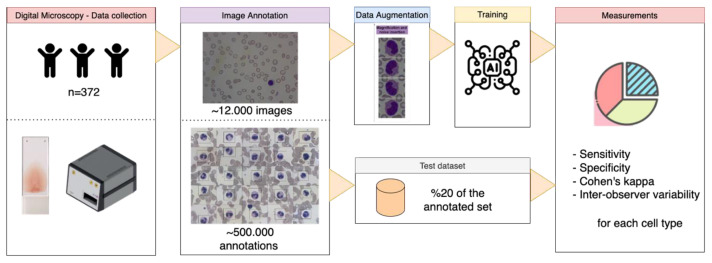
The workflow from the data collection to measurement metrics.

The main motivation of this study is to create a digital microscopy-based automated blood cell morphology analyzer for WBC with 10 subtypes (Basophil, Neutrophil, Lymphocyte, Monocyte, Eosinophil, Hypersegmented neutrophil, Band neutrophil, Myeloblast, Lymphoblast, and Reactive lymphocyte), RBC with 16 subtypes (Pencil cell, Stomatocyte, Microcytic RBC, Macrocytic RBC, Schistocyte, Bite cell, Spherocyte, Teardrop cell, Target cell, Knizocyte, Elliptocyte, Reticulocyte, Hypochromic RBC, Echinocyte, Sickle cell and Normoblast), platelets, platelet clumps, and artifacts recognition. During this study, Mantiscope, a cloud-based slide scanner system [12], has been used to collect images from PBS samples stained with Wright’s, in 100 x optical and 10 x digital magnification as a manual microscope with immersion oil. The quality (thickness of the blood film, staining features, color of the dye, etc.) of the PBS was checked by the laboratory technician and the hematologist to make sure the material was appropriate for Mantiscope scanning. Besides, if the PBS slide was found inadequate by the hematology expert during the annotation process, it is not labeled, and the case is changed to another sample. The PBS sample collection process was planned for the entire pediatric population under 18 years of age in the Pediatric Hematology-Oncology Department of Başkent University Adana Dr. Turgut Noyan Application and Research Center, and it lasted for 6 months. The dataset consists of 372 individuals, which 48 were normal and 324 had a specific hematological disorder (hereditary spherocytosis, thalassemia minor, thalassemia major, acute lymphoblastic leukemia, etc.,). An analyzable region is determined over the slide scanner to collect 12 × 12 counts in width and height respectively, equally spaced regions (~100mm × 75mm) from each slide. Each image contains an approximate number of 80 cells to be recognized. After digitizing the smear samples, physicians’ annotations, i.e. specifying a rectangle over cell objects by defining its type and boundary region, over the digital images are collected through the cloud-based software. The annotation platform is designed in a double-blind format to collect the interobserver variability across physicians. To develop an unbiased method by preventing the physicians from being influenced while annotating the contradictory cells in human perception, the annotators do not have any preliminary diagnostic information about individuals. In addition, they also do not follow other physicians’ annotations. As a result, the collected dataset used for training consists of only clear and visually recognizable cells. An approximate number of 500,000 annotations over 12,000 images were collected from three hematology experts having different roles to create the objective dataset (Table 1). While one of the hematology experts works in the full annotation process, the other two have annotation roles aiming creation of the dataset used for interobserver variability measurement. The workflow used in the annotation process for the organization can be seen in Figure 1.

### 2.2. Neural network, data augmentation, and learning

**Figure 2:**
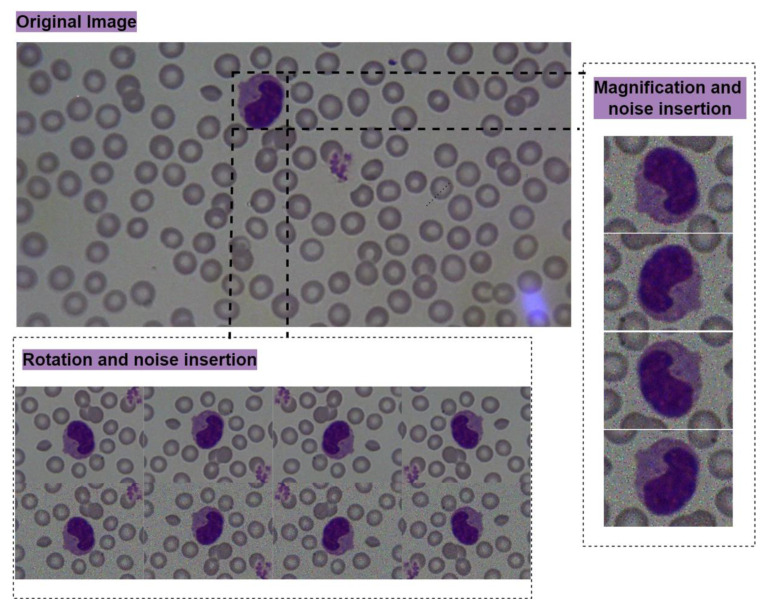
Data augmentation technique to create an effective learning methodology.

In order to develop a cell morphology analyzer using DL, state-of-the-art object detection using NN contains segmentation and classification tasks. Region proposals for the classification require extra effort for the processing units due to the possibility of having a large number of regions to be classified. Some of the NN architectures have data adaptive region proposal forms by using extra computing power [13,14]. Despite Faster R-CNN’s efficient object proposals and faster computations, it would not be preferable for smaller objects, such as platelets, in our case. Therefore, ‘You Only Look Once (YOLO)’ NN architecture is used for the detection of blood cells, because of having a region proposal model in pyramidal search that would promote different sizes of object detection and faster computations [15]. Yolov4-tiny is selected as the reference model by inserting a spatial attention module that provides extra information about cell-type positions over the images. For spatial attention, average-pooling and max-pooling operations are applied along the channel axis, and they are concatenated to generate another feature extractor for the cell types [16]. Spatial attention is significant for blood cell classification due to cell eliminations at the boundaries and making a comparison between the dimensions of RBCs. For example, physician examinations for the RBC would change according to their dimensions when compared to the other ones and they would be associated with macrocytic, microcytic, and normal cells. Therefore, the data augmentation step also consists of placing the neighboring cell objects that are differentiated from the targeting cell to be recognized by others for their dimensions. The technique also consists of the Gaussian noise insertion in different colors for increasing the recognition accuracy. The ground truth is randomly partitioned into test (20%) and training (80%) clusters for the measurement and training steps. This clustering ratio depends on obtaining a feasible number of cell types over the test set. We have detected this ratio to have a minimum number of 100 annotations for each cell type testing. The training cluster is used for transferring the domain expertise into the DL algorithm. The full training data is partitioned into 64 images per batch and 16 images for chunks. The proposed learning method for the cell morphology analyzer is data augmentation to increase the diversity of the annotated dataset. As the micro view, a single cell over a PBS slide can be positioned in different perspectives, and although it can contain noisy regions, it would be identified by a domain expert with a microscope while performing the manual evaluation process. Therefore, on behalf of a single-cell object representation, a group of cell object images is generated to be useful during training. Figure 2 represents the base cell object and its generated images used during training.

### 2.3. Measurement methods

The method to measure objective metrics, sensitivity, and specificity depends on updating the probability threshold and Intersection over Union (IOU) parameters, which are used to identify and associate the detected object with the ground truth by the DL algorithm. While the training cluster is only used for transferring the expertise into the neural network (NN), the testing cluster (20% of the full set) is used for evaluating the NN using IOU and probability thresholds, which are selected as 0.5 and 0.25, respectively. Test cluster images are analyzed with the developed DL algorithm, and the prediction results are compared with the ground truth. If the observed IOU represents the intersection ratio of the possible ground truth with the prediction result, the observed probability threshold is greater than defined thresholds and their cell types are the same, it is labeled as True Positive (TP). If IOU satisfies the desired criterion, but the cell type differs from the ground truth, it is labeled as False Positives (FP). If the IOU does not satisfy the criterion, it is labeled as False Negative (FN). Measured True Negatives (TN) for each cell are calculated by summing the TP of the negatives associated with each cell type given in Table 1. The equations for sensitivity, specificity, and Cohen’s kappa metrics are given with the help of TP, TN, FP, and FN [17,18]. We have increased the number of cells related to the negative sets of each cell type to improve the specificity. In addition, Cohen’s kappa is a quantitative measurement of reliability between two evaluators rating the same variable. A score of 1 means that there is a complete agreement between the evaluators [18]. Precision is also defined as accuracy. It is important when false positives are costly. Lower precision means diagnosing a disease when someone does not have it. F1-score is also a measure of the balance between sensitivity and precision. It is used to validate the model’s effectiveness [19].


(1) $$\text{Sensitivity}=\frac{TP}{TP+FN}$$

(2) $$\text{Specificity}=\frac{TN}{TN+FP}$$

(3) $$\text{Cohen}’\text{s kappa}=\frac{2*(TP*TN-FP*FN)}{\left(TP+FP)*(FP+TN)+(TP+FN)*(TN+FN\right)}$$

(4) $$\text{Precision}=\frac{TP}{TP+FP}$$

(5) $$\text{F}1\text{-score}=\frac{2X\;Precision\;X\;Recall\;(Sensitivity)}{Precision\;+\;Recall\;(Sensitivity)}$$

Subjective measurements are also performed between three evaluators to measure the correlation coefficient. It is used to observe the interobserver variability between physicians. Twenty randomly selected PBS digital images which are not previously used for AI training are annotated in a double-blind format, and physicians’ annotations are compared with an IOU threshold of 0.5. The primary evaluator’s annotations are chosen as a reference for the comparison. The correlation coefficient is calculated by dividing the positive correlation by the sum of positive and negative correlations.

## 3. Results

**Figure 3:**
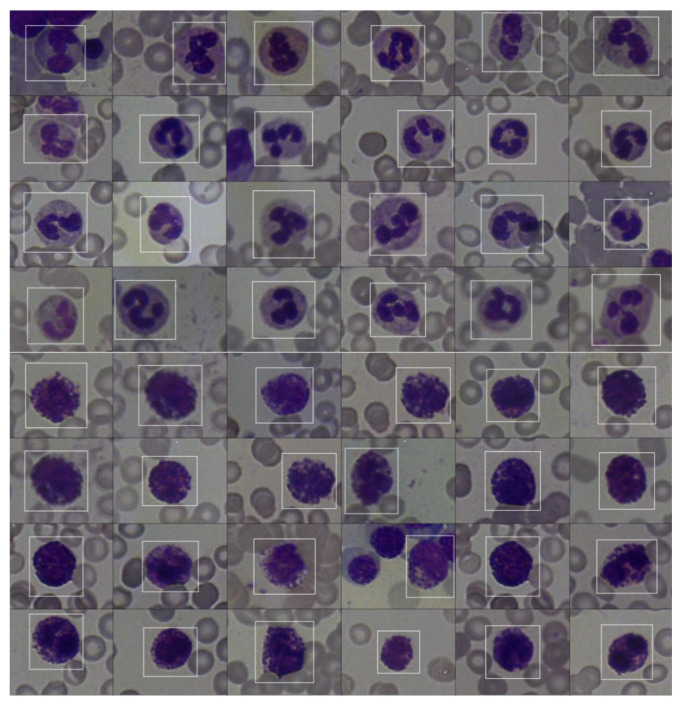
An example of a set of annotated cell groupings for neutrophil and basophil.

According to the annotation results, we have obtained an unbalanced condition that occurs in the cell counts due to the incidence differing in the cell types. Therefore, augmentation methods seen in Figure 2, such as mirroring, and hue/saturation/exposure parameter variances are used to create a balanced dataset to use in training DL networks. The resulting cell counts obtained after annotation and augmentation can be seen in Table 1. For example, the number of microcytic RBCs that were labeled the most is 188,246, the total number obtained after annotation is 941,230 and spherocytes, macrocytic RBCs and hypochromic RBCs, which are likely to be morphologically similar to microcytic RBCs, are selected as negative ones. The common classified features of WBC can be identified with cytoplasm/nucleus ratio, cytoplasm colors, cytoplasmic granules and segmentation type of the nucleus during the manual microscopy process. Although these features can be defined objectively in manual microscopy, the cells from the digitized images cannot be identified easily due to digital magnification and pixelization effects. Therefore, interobserver variability is an important metric for creating the positive/negative samples and each positive cell type is also associated with the negative sets to measure the specificity. According to the interobserver variability metric, Table 1 is given to specify the cell grouping (positive/negative) for measuring the method’s specificity. This information is used to narrow the negative set instead of using the full cluster. A group of samples comprising annotated cells can also be seen in Figure 3.

By evaluating the learning loss and mean average precision (mAP) during training, we have selected the trained weights captured when these metrics become stable for measurements. This has been achieved to prevent the NN from memorizing the results instead of learning because training the NN after this point would decrease the measurement results. The developed decision support system is verified using randomly selected and partitioned ground truth from the annotated dataset. Sensitivity and specificity are measured for each cell type, and they are reported in Table 2. From the view of specificity, we obtained values higher than 92% in the entire study group. The sensitivity was also above 80%. If we look at the cell subgroups in detail, WBCs, myeloblasts, and basophils had been defined by NN by 98% and 94% sensitivity and 98% and 99% specificity, respectively. Hypersegmented neutrophils and reactive lymphocytes had the lowest sensitivity, 68%, and 73% respectively. For RBCs, normoblasts, pencil cells and target cells had been defined by NN by 93%, 81%, and 81% sensitivity and 99%, 99%, and 79% specificity, respectively. The lowest sensitivity was obtained in bite cells and echinocytes (47% vs. 61%), while the lowest specificity was obtained in elliptocytes and microcytic RBCs (42% vs. 55%). In sensitivity and specificity measurements of platelets, values above 70% were obtained. If Cohen’s kappa measurements are evaluated, the highest values for WBCs were obtained in basophils, and band neutrophils (0.92 vs 0.91), while for RBCs, the highest values were obtained in normoblasts, sickle cells, and pencil cells (0.89, 0.78 vs. 0.77). When the precision parameters are evaluated, they are greater than 0.5 for each cell type excluding the Bite cell. It seems that the algorithm is unlikely to miss a patient having disorders. F1-score values are also higher than 0.6 which shows the balance between accuracy and sensitivity (Table 2).

The results given in Table 2 consist of measurement metrics obtained after data augmentation. The learning methodology proposed in this study is also tested by training the system without data augmentation. In other words, only the annotations collected from the physicians are used during training. It is observed that while the results obtained through data augmentation outperform in the classification of WBC types, a significant improvement cannot be obtained through RBC types. The results obtained without data augmentation for basophil, neutrophil, lymphocyte, eosinophil, and hypersegmented neutrophil are given as 0,82, 0.81, 0.82, 0.79, and 0.60, respectively.

Subjective measurements performed between three hematology experts also reveal that there is not a strict correlation between decision-makers if digital microscopy images are used for blood cell morphology evaluation. The correlation calculated between the physicians’ evaluationsis given in Table 3 representing the interobserver variability. While the physicians can identify neutrophils, lymphocytes, pencil cells, spherocytes, and echinocytes in a strong correlation, there is not a strong correlation to identify the other cell types.

## 4. Discussion

Peripheral blood smear findings of several diseases are distinctive and it provides morphological findings to clinicians with significant convenience in terms of rapid diagnosis and avoiding delays in treatment and follow-up. For example, PBS findings of nutritional anemias are hypochromia, microcytosis, ovalocytes, tear drop cells and in severe cases target cells and pencil cells for iron deficiency, and macrocytic RBC, macroovalocytes, tear drop cells, increased number of hypersegmented neutrophils for megaloblastic anemia. Common PBS findings for hemolytic anemias are target cells, tear drop cells, ovalocytes, stomatocytes, normoblasts, reticulocytes, schistocytes, spherocytes or sickle cells according to specific underlying disorders. In many acute leukemia cases, blasts can be seen over PBS. In terms of initial rapid and accurate diagnosis, a pre-trained cell-morphology analyzer machine would be helpful.

There are some challenges for physicians having intense conditions in urban areas to obtain PBS. In addition, due to technical problems, the workflow for the diagnostics may not progress quickly. In the awareness of these problems, researchers have tried to develop digital systems over the years, and AI-assisted microscopes have emerged. Kratz et al., Bachar et al., Rodellar et al., Jiang et al., Hedge et al., and many other researchers evaluated AI algorithms they developed and brought them to the literature [20–24]. In this process, we have observed from the WBC measurement table that we would obtain sensitivity greater than 80% except for the hypersegmented neutrophils and reactive lymphocytes. False negative is high for these cell types because neural networks have identified these types of cells as neutrophils and lymphocytes. It is probably related to the unbalanced condition which is obtained between the main types and subtypes. Although the false negative rates for hypersegmented neutrophils and reactive lymphocytes are not significantly considered in terms of malignant diseases, the real problem is the false positive rates of lymphoblasts and myeloblasts. This was a point that caught our attention in the results. However, we think that our study is important in terms of emphasizing that it would not be a wrong decision to have the peripheral smear evaluations of these patients performed by a hematologist at the decision stage and that the machine should not be used for diagnosis and treatment alone. In addition, while screening the literature, it is seen that similar results were obtained in other studies. From the view of specificity, we would also obtain values higher than 90% in all WBC types. CellaVision, an automated digital image analyzer, pre-classification accuracy was reported as 89.2% for overall WBC subgroups, but poor detection rates were reported for eosinophils, band neutrophils and basophils [25,26]. Another well-known digital cell image analyzer, DI-60 system (Sysmex) was good enough for WBC classification [27]. However, there were some differences between the DI-60 and manual counting for RBC evaluations [27,28]. In a study comparing WBC subgroups between DI-60 and Mindray MC-80 which is a new morphology analyzer, the sensitivity for reactive lymphocytes was 28.6% with MC-80 and 100% with DI-60, while the sensitivity for blasts was 92% with MC-80 and 49.3% with DI-60 [29]. In the study where immature neutrophils were analyzed with 3 different digital systems (CellaVision DM96, CellaVision DM100, iCELL ME-150), sensitivity and specificity were found significantly high with all three models (83.5% vs. 96.9%) [30]. With the algorithm published in the article, whole blood cells of healthy individuals were analyzed and over 95% sensitivity and specificity were obtained for eosinophils, lymphocytes, and monocytes in the WBC types [31].

Although there are many studies on WBC types, there are fewer studies on morphology analysis of RBC series. Horn et al reported variable sensitivity rates in erythrocytes with CellaVision, reaching 100% for sickle cell and stomatocytes [32]. In addition, Huisjes et al. reported that they consider CellaVision as a useful screening tool for hereditary hemolytic diseases [33]. Foy et al. achieved 72% specificity and 94% sensitivity for schistocytes in all selected RBC types, with a machine-learning approach called RBC-diff [34]. In our study, we would obtain sensitivity values greater than 80% for normoblast, pencil cell, target cell, and sickle cell when RBC measurements are evaluated. But for some cell types like bite cells, sensitivity was low. Their FN is higher when compared to TP. This would be related to a lower number of annotation counts for these cell types. When we compare the specificity, we observe that microcytic RBC and elliptocyte are lower, and we would observe specificity values greater than 95% for normoblast, pencil cell, bite cell, spherocyte, tear drop cell, echinocyte, and sickle cell. It should be noted that since the evaluation process is handled between the physicians and AI predictions, lower specificity does not mean the system recognizes some cell types in the wrong classification. Finally, we reached over 80% sensitivity and specificity for platelet clumps in our study. For the corresponding cell type, 50% sensitivity has been reported by CellaVision [35,36].

Annotation-based digital evaluation of deep learning algorithms discloses the objective metrics by the percentage of sensitivity and specificity. However, morphology-based diagnostics for PBS samples by manual microscopy vary among observers via resulting in unpredictable intra- and interobserver variability. The standard evaluation steps comprise counting blood cells in an evaluation window with different morphological features and estimating each blood cell type percentage. While it is a manual process, counting cells accurately using ML techniques in a higher number of regions would improve the results by increasing the number of images collected through the samples. According to the results of our study, it is observed that there is not strict correlation (1.0) between hematologists when the images are evaluated in detail from Table 3. Besides, physicians could not identify the WBC subtypes in a strong correlation. Therefore, we can conclude that neural networks can classify WBC morphologies from a large variety of cells in an acceptable correlation with physicians. In other words, cell morphology analysis would differentiate concerning the physicians’ expertise. Therefore, an automated blood cell morphology analyzer with a measured sensitivity and specificity can be used as a prediagnostics decision support system to create alerts for the physicians and it can be used as an assistant. In a study conducted by Xing et al., technologists having three different experience periods were compared and the accuracy rates in the evaluation of WBCs increased significantly and the average evaluation times were significantly shortened in cases assisted by AI as per manual microscopy [37]. Briefly, studies reveal that it would be possible to transfer patients to hematology departments earlier by general practitioners with the help of AI systems.

When the articles in the literature are reviewed, our study is one of the rare uniform studies that analyze whole blood cells rather than searching for a specific cell disorder and children aged 18 years and younger were only included. Furthermore, this paper illustrates the study generated within 6 months of data collection of 372 cases and annotation of the smears of these children by three physicians. Through this process, a specificity of over 90% had been obtained with main cell groups. Since the annotation and AI development process and machine learning are a continuous process, we may collect more data from more individuals and capture different evaluations from other physicians’ expertise, we would obtain higher sensitivity and specificity for associated cell types and this study should be extended for bone marrow examination. New training techniques and DL architectures will also be discussed to get more accurate classification results for future work. Finally, as the hematology analyzers used in the laboratories provide analytics for a limited number of cells, we aim to create a comprehensive cell analysis algorithm also containing red blood cells in this study.

## Data availability statement

The data that support the findings of this study are available from the corresponding author upon reasonable request.

## Figures and Tables

**Table 1:** WBC, RBC, PLT annotation counts, and their negative clusters used for this study.

Cell Type (Positive)	w. Augmentation	Annotations	Total	Cell Type (Negative)
Basophil	664	166	830	Lymphocyte, Neutrophil
Neutrophil	28,348	7087	35,435	Eosinophil, Monocyte, Lymphocyte, Basophil
Lymphocyte	28,648	7162	35,810	Neutrophil, Monocyte, Basophil, Eosinophil, Lymphoblast, Reactive lymphocyte, Myeloblast
Monocyte	3104	776	3880	Lymphoblast, Reactive lymphocyte, Lymphocyte
Eosinophil	2088	522	2610	Neutrophil, Band neutrophil, Hypersegmented neutrophil
Hypersegmented neutrophil	744	186	930	Neutrophil, Band neutrophil, Eosinophil
Band neutrophil	1988	497	2485	Neutrophil, Hypersegmented neutrophil, Eosinophil
Myeloblast	868	217	1085	Lymphocyte, Lymphoblast, Monocyte
Lymphoblast	5624	1406	7030	Reactive lymphocyte, Lymphocyte, Myeloblast, Monocyte
Reactive lymphocyte	3524	881	4405	Lymphoblast, Lymphocyte, Myeloblast, Monocyte
Pencil cell	2976	744	3720	Sickle cell, Elliptocyte
Stomatocyte	40,084	10,021	50,105	Elliptocyte
Microcytic RBC	752,984	188,246	941,230	Spherocyte, Macrocytic RBC, Hypochromic RBC
Macrocytic RBC	34,012	8503	42,515	Reticulocyte, Microcytic RBC, Hypochromic RBC
Schistocyte	6552	1638	8190	Bite cell
Bite cell	3472	868	4340	Spherocyte, Schistocyte
Spherocyte	43,788	10,947	54,735	Macrocytic RBC, Bite cell
Teardrop cell	7900	1975	9875	Elliptocyte
Target cell	25,400	6350	31,750	Stomatocyte, Knizocyte
Knizocyte	5724	1431	7155	Target cell
Elliptocyte	181,604	45,401	227,005	Teardrop cell, Stomatocyte
Reticulocyte	8040	2010	10,050	Spherocyte, Macrocytic RBC
Hypochromic RBC	176,868	44,217	221,085	Spherocyte
Echinocyte	7784	1946	9730	Spherocyte, Macrocytic RBC, Microcytic RBC
Sickle cell	2412	603	3015	Bite cell
Platelets	84,968	21,242	10,6210	Artifacts, Platelet clump
Platelet clump	41,832	10,458	52,290	Platelets, Artifact
Normoblast	1424	356	1780	Lymphocyte, Reactive lymphocyte, Myeloblast, Lymphoblast
Artifact	10,128	2532	12,660	Platelets, Platelet clump

**Table 2:** Measurements for the WBC, RBC, platelets, platelet clump, and artifact.

Cell Type	TP	FP	TN	FN	Sensitivity	Specificity	Cohen’s Kappa	Precision	F1-score
Basophil	131	12	9453	9	0.94	0.99	0.92	**0.92**	**0.93**
Neutrophil	4641	499	5772	951	0.83	0.92	0.75	**0.9**	**0.86**
Lymphocyte	4812	575	7299	917	0.84	0.93	0.77	**0.89**	**0.86**
Monocyte	509	75	6350	90	0.85	0.99	0.85	**0.87**	**0.86**
Eosinophil	320	23	5117	71	0.82	0.99	0.86	**0.93**	**0.87**
Hypersegmented neutrophil	110	14	5079	52	0.68	0.99	0.76	**0.89**	**0.77**
Band neutrophil	366	33	5071	37	0.91	0.99	0.91	**0.92**	**0.91**
Myeloblast	160	89	6362	4	0.98	0.98	0.77	**0.64**	**0.77**
Lymphoblast	1041	495	5978	116	0.90	0.92	0.73	**0.68**	**0.77**
Reactive lymphocyte	497	48	6522	184	0.73	0.99	0.79	**0.91**	**0.81**
Pencil Cell	492	168	26,699	116	0.81	0.99	0.77	**0.75**	**0.78**
Stomatocyte	5848	2247	26,699	2275	0.72	0.92	0.64	**0.72**	**0.72**
Microcytic RBC	117,604	49,006	59,759	39,202	0.75	0.55	0.30	**0.71**	**0.73**
Macrocytic RBC	4838	3372	153,141	1790	0.73	0.98	0.64	**0.59**	**0.65**
Schistocyte	875	535	9951	412	0.68	0.95	0.60	**0.62**	**0.65**
Bite cell	310	393	8136	350	0.47	0.96	0.41	**0.44**	**0.45**
Spherocyte	7261	3717	122,565	2293	0.76	0.97	0.68	**0.66**	**0.71**
Teardrop cell	1120	753	26,699	480	0.70	0.97	0.62	**0.6**	**0.65**
Target cell	4117	1800	6626	966	0.81	0.79	0.58	**0.7**	**0.75**
Knizocyte	778	285	4117	334	0.70	0.94	0.65	**0.73**	**0.71**
Elliptocyte	26,699	12443	8934	9875	0.73	0.42	0.15	**0.68**	**0.7**
Reticulocyte	1428	916	12,099	357	0.80	0.93	0.64	**0.61**	**0.69**
Hypochromic RBC	23,625	13,268	153,738	15,105	0.61	0.92	0.54	**0.64**	**0.62**
Echinocyte	1093	573	124,865	699	0.61	0.99	0.63	**0.66**	**0.63**
Sickle cell	386	112	27191	97	0.80	0.99	0.78	**0.78**	**0.79**
Normoblast	309	47	7163	24	0.93	0.99	0.89	**0.87**	**0.9**
Platelet	13,239	6125	16,744	3955	0.77	0.73	0.49	**0.68**	**0.72**
Platelets clump	6877	1667	13,465	1614	0.81	0.89	0.70	**0.8**	**0.8**
Artifact	1710	181	772	279	0.86	0.81	0.65	**0.9**	**0.88**

**Table 3:** Interobserver variability measurements between primary annotator (PA), first annotator (A1) and second annotator (A2).

Cell Type	Positive Correlation (PA-A1)	Negative Correlation (PA-A1)	Correlation Coefficient (PA-A1)	Positive Correlation (PA-A2)	Negative Correlation (PA-A2)	Correlation Coefficient (PA-A2)
**Neutrophil**	17	2	**0.89**	19	0	**1.00**
**Lymphocyte**	7	0	**1.00**	6	1	**0.86**
**Neutrophilband**	0	2	**0.00**	0	2	**0.00**
**Reactivelymphocyte**	0	1	**0.00**	0	1	**0.00**
**Pencil cell**	12	3	**0.80**	9	6	**0.60**
**Stomatocyte**	14	43	**0.25**	27	30	**0.47**
**Microcytic RBC**	447	472	**0.49**	358	561	**0.39**
**Macrocytic RBC**	20	39	**0.34**	16	43	**0.27**
**Schistocyte**	5	1	**0.83**	5	1	**0.83**
**Bite cell**	1	1	**0.50**	2	0	**1.00**
**Spherocyte**	43	6	**0.88**	42	7	**0.86**
**Teardrop cell**	11	22	**0.33**	18	15	**0.55**
**Target cell**	116	12	**0.91**	92	36	**0.72**
**Knizocyte**	2	7	**0.22**	7	2	**0.78**
**Elliptocyte**	380	132	**0.74**	264	248	**0.52**
**Reticulocyte**	3	17	**0.15**	13	7	**0.65**
**Hypochromic RBC**	360	333	**0.52**	425	268	**0.61**
**Echinocyte**	1	0	**1.00**	0	1	**0.00**
**Sickle cell**	10	2	**0.83**	9	3	**0.75**
**Platelets**	35	38	**0.48**	51	22	**0.70**
**Platelet clump**	15	23	**0.39**	27	11	**0.71**
**Artifact**	2	4	**0.33**	0	6	**0.00**
